# Hypothermic Oxygenated New Machine Perfusion System in Liver and Kidney Transplantation of Extended Criteria Donors:First Italian Clinical Trial

**DOI:** 10.1038/s41598-020-62979-9

**Published:** 2020-04-08

**Authors:** Matteo Ravaioli, Vanessa De Pace, Andrea Angeletti, Giorgia Comai, Francesco Vasuri, Maurizio Baldassarre, Lorenzo Maroni, Federica Odaldi, Guido Fallani, Paolo Caraceni, Giuliana Germinario, Chiara Donadei, Deborah Malvi, Massimo Del Gaudio, Valentina Rosa Bertuzzo, Antonio Siniscalchi, Vito Marco Ranieri, Antonietta D’Errico, Gianandrea Pasquinelli, Maria Cristina Morelli, Antonio Daniele Pinna, Matteo Cescon, Gaetano La Manna

**Affiliations:** 1 Department of General Surgery and Transplantation, University of Bologna Sant’Orsola - Malpighi Hospital, Bologna, Italy; 2 Department of Experimental Diagnostic and Specialty Medicine, University of Bologna Sant’Orsola- Malpighi Hospital, Bologna, Italy; 3 Department of Medical and Surgical Science, University of Bologna Sant’Orsola - Malpighi Hospital, Bologna, Italy

**Keywords:** Clinical trial design, Kidney, Kidney diseases

## Abstract

With the aim to explore innovative tools for organ preservation, especially in marginal organs, we hereby describe a clinical trial of *ex-vivo* hypothermic oxygenated perfusion (HOPE) in the field of liver (LT) and kidney transplantation (KT) from Extended Criteria Donors (ECD) after brain death. A matched-case analysis of donor and recipient variables was developed: 10 HOPE-ECD livers and kidneys (HOPE-L and HOPE-K) were matched 1:3 with livers and kidneys preserved with static cold storage (SCS-L and SCS-K). HOPE and SCS groups resulted with similar basal characteristics, both for recipients and donors. Cumulative liver and kidney graft dysfunction were 10% (HOPE L-K) *vs*. 31.7%, in SCS group (*p* = *0.05*). Primary non-function was 3.3% for SCS-L *vs*. 0% for HOPE-L. No primary non-function was reported in HOPE-K and SCS-K. Median peak aspartate aminotransferase within 7-days post-LT was significantly higher in SCS-L when compared to HOPE-L (637 *vs*.344 U/L, *p* = *0.007*). Graft survival at 1-year post-transplant was 93.3% for SCS-L vs. 100% of HOPE-L and 90% for SCS-K vs. 100% of HOPE-K. Clinical outcomes support our hypothesis of machine perfusion being a safe and effective system to reduce ischemic preservation injuries in KT and in LT.

## Introduction

The gap between patients candidate to liver or kidney transplant and the actual organ availability is a prominent issue for transplant centres worldwide, and has a significant impact for such patients in terms of morbidity and mortality^1,2^. Nevertheless, the number of discarded organs is increasing constantly, primarily as a consequence of the increasing average age of the donor pool^1^.

Several data demonstrate how the use of marginal liver and kidney allografts, defined according to the Extended Criteria Donors(ECD), may represent a valid strategy to enlarge the donor pool and to offer more transplant opportunities otherwise unachievable only with Standard Criteria Donors (SCD)^3–6^. However, the use of ECD organs still entails a considerable price to pay: an increase of primary non-function (PNF) or delayed graft function (DGF) in kidney transplant (KT) and early allograft dysfunction (EAD) in liver transplant (LT), and a decrease of long-term survival^7^ not related to the experience of the transplant centers^8^. Cold ischemia time (CIT) and warm ischemia time during reperfusion impair ECD grafts – being those highly vulnerable organs – but at the same time do not increase mortality risk, which remains relevant for patients in waiting list^9^. These evidences lead scientific transplant community to explore new strategies for the preservation of marginal grafts, which appear more vulnerable when treated with standard techniques, such as static cold storage (SCS). Different innovative preservation methods in KT and LT are currently under investigation in animal models, pre-clinical and clinical trials, comparing different strategies, ranging from hypothermic (4–10 °C) to subnormothermic (20–25 °C) – including controlled oxygenated rewarming – and normothermic (35–37 °C) conditions^10^. Early clinical evidences from the Zurich group support the benefit of hypothermic oxygenated perfusion (HOPE) as confirmed for LT clinically^11^ and on rat models for KT experimentally^12^. Conversely, in United Kingdom, normothermic machine perfusion (NMP) for preservation of LT and KT achieved an optimal safety, feasibility and efficacy^13,14^. Subnormothermic machine perfusion (SMP) and controlled oxygenated rewarming (COR) were experienced in animal models demonstrating the improvement in renal or liver function and superiority when compared to SCS^15–17^, but to present only COR in LT was applied in clinical practice with good results of feasibility and short-term transplant outcomes^18^.

Our preliminary experimental study on human discarded kidneys and liver grafts^19^ lead us to confirm the role of dynamic preservation and the need for oxygen provision to restore ATP levels, despite the fact that their clinical marginal features had labeled such organs as un-transplantable. Therefore, based on our previous findings and several positive suggestions above mentioned^11,12^, we designed an interventional clinical trial to apply HOPE as preservation technique in liver and kidney transplant procedure. HOPE was performed through a machine perfusion purposely developed for abdominal organs perfusion in our center. Outcomes data were elaborated to determine safety and efficacy of HOPE treatment in extended criteria donors after brain death (ECD-DBD) for LT and KT.

## Results

### Donor and recipient characteristics

Between October 2016 and December 2017, N = 20 recipients (N = 10 LT recipients and N = 10 KT recipients) were recruited consecutively and transplanted with ECD-DBD organs preserved from surgical back-table to implantation by HOPE (Table 1). The main indication for LT was hepatocellular carcinoma on cirrhosis with one or more etiologies: alcoholic liver disease (N = 2), hepatitis C virus infection (N = 5), hepatitis B and D virus infection (N = 2), hepatitis B virus infection (N = 1) and primary biliary cirrhosis (N = 1); only one patient was affected by alcoholic cirrhosis only. Recipients of KT had end-stage renal disease with the following etiologies: interstitial nephritis (N = 1), renal polycystosis (N = 2), hypertensive renal vascular disease (N = 1), diabetic nephropathy (N = 1), hypertensive nephrosclerosis (N = 2), membranous glomerulonephritis (N = 1), nephroangiosclerosis (N = 1) and chronic glomerulonephritis (N = 1).

**Table 1:** Demographic and clinical data for liver and kidney matching analyses.

	Sperimental Group	Control group	P value
	HOPE	SCS	
*Liver Transplantation*	*N* = 10	*N* = 30	
Donor Age	77.5 (60–84) years	75.5 (53–85) years	0.396
Recipient Age	57.5 (50–68) years	60.5 (48–68) years	0.331
Cold Ischemia Time	7.1 (6.1–9.6) hours	7 (5.4–10) hours	0.528
MELD score	13 (7–16)	13.5 (7–20)	0.963
Previous abdominal surgery	5 (50%)	13 (43.3%)	0.681
Portal thrombosis	1 (10%)	3 (10%)	1
*Kidney Transplantation*	*N* = 10	*N* = 30	
Donor Age	71.5 (60–78) years	69.5 (59–79) years	0.653
Recipient Age	61 (50–65) years	60.5 (48–68) years	0.851
Cold Ischemia Time	14.5 (10.8–22) hours	14 (8–21) hours	0.896
Karpinsky’s score median	4	3	0.105
**Type of Dialysis**			
Peritoneal dialysis	4 (40%)	9 (30%)	0.492
Hemodialysis	6 (60%)	21 (70%)	
Dialysis Time	47.3 (19.6–108) months	47.3 (7.6–139.2) months	

Hypothermic Oxygenated Perfusion (HOPE) versus Static Cold Storage (SCS) in the liver and kidney transplantation. The values are expressed as median and range or number and percentage. No differences were found between the two groups.

The study population was properly matched with the control group (Table 1) and no significative differences were reported between the two groups.

Median donor age was very extended: 77.5 (60–84) years for HOPE-L group and 71.5 (60–78) yearsfor HOPE-K group compared to 75.5 (53–85) years for SCS-L group and 69.5 (59–79) years for SCS-K group (p = n.s.).

Median recipient age was 57.5 (50–68) years for HOPE-L group and 61 (50–66) years for HOPE-K group versus 60.5 (48–68) years for SCS-L group and SCS-K group (p = n.s.).

In the L-HOPE group, the median CIT was 7.1 (6.1–9.6) hours: a value time comparable to 7 (5.4–10) of L-SCS group and very low because the perfusion treatment started at the time of the organ back-table and the livers were immediately transplanted.

The median CIT for the kidney group was about 14 hours, partly due to the fact that we do not perform KT during the night in our centre. However, this data was still comparable among the HOPE-K and SCS-K groups, 14.5 (10.8–22) hours versus 14 (8–21) hours respectively.

Furthermore, L-HOPE and L-SCS groups were matched similarly for MELD score, previous abdominal surgery and portal thrombosis.

In the HOPE-K group, one patient receiving double KT was matched with 3 double KT cases in the control group.

The median of Karpinsky’s score was 3 for SCS-K group and 4 for HOPE-K group. Type and time of dialyses were also equally distributed between study and control group.

### Histopathological features

The liver histology of the study groups are reported in Supplementary Materials Table 1, where nine histological parameters were evaluated in HOPE-L and SCS-L. No statistically significant differences were reported.

Kidney histology are reported in Supplementary Materials Table 2: thirteen histological variables were evaluated in HOPE-K and SCS-K. No statistically significant differences were found between the two groups.

### Perfusion parameters and biochemical characteristics of the HOPE perfusates

The perfusion parameters of N = 11 kidneys, N = 9 for single KT and N = 2 for dual KT, and N = 10 livers undergoing HOPE before the implantation are reported in Table 2. No adverse events during organ perfusion were registered for study cases enrolled.

**Table 2:** Machine perfusion and biochemical data before (T0) and after (T1) hypothermic oxygenated perfusion (HOPE).

Organ	Flow	Pressure	Resistence	Temperature	Time	pH T0	pCO_2_ T0	paO_2_ T0	Lat T0	pH T1	pCO_2_ T1	paO_2_ T1	Lat T1
1_Kidney	55	30	0.54	4	60′	6.85	8	346	5.4	<6.8	9	647	9
2_Kidney	52.5	30	0.57	4	200′	6.88	7	204	4.5	6.83	7	706	11.7
3_Kidney	29	30	1.03	4	100′	<6.8	6	483	<2.7	<6.8	6	688	6.3
4_Kidney	46	30	0.65	4	360′	6.83	<6	779	<2.7	6.82	6	732	13.5
5_Kidney	30	30	1	4	240′	6.87	<6	112	<2.7	<6.8	6	784	6.3
6_Kidney	55	30	0.54	4	270′	6.86	<6	93	<2.7	6.82	8	778	12.6
7_Kidney	60	30	0.5	4	300′	7.03	7	185	3.6	6.94	8	718	11.7
8_Kidney	85	30	0.35	4	150′	7.17	<6	172	8.1	7.17	<6	723	22.5
9_Kidney	52	30	0.57	4	150′	6.82	<6	191	3.6	<6.8	6	741	10.8
10_Kidney	24	30	1.25	4	80′	<6.8	6	546	9.9	6.93	8	623	10
11_Kidney	77	30	0.32	4	205′	7.27	<6	132	<2.7	7.23	<6	675	12.6
1_Liver	25	5	0.2	4	75′	<6.8	6	78	3.6	<6.8	10	632	16.2
2_Liver	116	5	0.04	4	60′	6.88	8	155	6.3	6.84	8	688	10.8
3_Liver	110	5	0.04	4	170′	<6.8	9	190	8.1	<6.8	10	714	14.4
4_Liver	114	5	0.04	4	115′	<6.8	12	225	17.1	<6.8	11	582	24.3
5_Liver	100	5	0.05	4	165′	6.83	<6	105	2.7	6.83	6	706	9.9
6_Liver	109	5	0.04	4	130′	6.88	<6	123	<2.7	6.95	8	685	19
7_Liver	100.5	5	0.05	4	135′	6.86	<6	265	<2.7	6.83	10	605	9
8_Liver	106	5	0.05	4	210′	6.88	13	106	14.4	6.96	9	653	19.8
9_Liver	65	5	0.07	4	90′	7.23	<6	204	8.1	7.14	7	678	27
10_Liver	30	5	0.16	4	160′	7.18	9	155	26.1	7.31	8	682	2.7

pH = −log_10_ hydrogen ion concentration in moles per liter; pCO_2_ = partial pressure of carbon dioxide; PaO_2_ = partial pressure of Oxygen; Lat = lactate.

The median hepatic portal flow was 107.5 (65–116) mL/min and median perfusate lactate levels was 16.2 (9–27) mg/dL, as reported in Table 3.

**Table 3:** Perfusion data of liver and kidney dynamic preservation. The values are expressed as median and range.

	Sperimental Group
	HOPE
*Liver HOPE*	*N* = 10
Vein Portal Flow	107.5 (65–116) mL/min
Lactate post HOPE	16.2 (9–27) mg/dL
Perfusion Time	2.2 (1–3.5) hours
*Kidney HOPE*	*N* = 10
Renal Flow	52.5 (24–85) mL/min
Lactate post HOPE	11.7 (6.3–22.5) mg/dL
Perfusion Time	3.3 (1–6) hours

The median renal flow was 52.5 (24–85) mL/min and median perfusate lactate levels was 11.7 (6.3–22.5) mg/dL, as reported in Table 3.

Oxygen level was in line to the reference limits during HOPE as demonstrated by the paO_2_ values at T4 in the Table 2.

The mean perfusion time was 2.2 (1–3.5) hours for HOPE-L group and 3.3 (1–6) hours for HOPE-K group. We had only 2 cases of single KT whose perfusion time was lower than 2 hours, while all other cases had a longer perfusion time.

Bile or urine production were not assessed during the liver or kidney perfusion treatment, respectively.

Microbiological analysis of the perfusate specimens did not evidence any bacterial or fungal growth defined as pathogen contamination and related to the perfusion strategy under study.

### Graft function and post-transplant complications

Variables related to graft dysfunction were showed in Table 4. Overall graft dysfunction, defined as EAD in the HOPE-L and DGF in the HOPE-K groups, was 10% *vs*. 31.7% in the control group, p = 0.05. Primary non-function was 0% for both K-groups and 6.6% for SCS-L *vs*. 0% for HOPE-L, p = 0.086.

**Table 4:** Clinical outcomes of hypothermic oxygenated perfusion and control organs.

Kidney	HOPE (N = 10)	Control (N = 30)	Odds ratio/Effect size (CI interval)	p value
PNF, n (%)	0 (0%)	1 (3.3%)	0.9667 OR (0.3506–2.6656)	0.9478
***DGF***, ***n (%)***	***2 (20%)***	***12 (40%)***	0.7500 OR (0.2501–2.2489)	0.6076
Creatinine at 5 day post-transplant, media (SD)	3.5 ± 2	4.1 ± 2.6	− 0.24 ES (−0.96 to 0.48)	0.4572
eGFR on dimission day (mL/min/1.73 m²), media (SD)	52.4 ± 25.01	44.43 ± 21.4	− 0.35 ES (−0.37 to 1.07)	0.3762
Hospital stay (days), median (range)	17 (12–30)	24 (11–60)	− 0.49 ES (−1.22 to 0.23)	0.0924
***1***-***year graft survival***, ***n (%)***	***10 (100%)***	***28 (93.3%)***	0.9333 OR (0.3377–2.5796)	0.8936
***1-year recipient survival***, ***n (%)***	***10 (100%)***	***29 (96.6%)***	0.9667 OR (0.3506–2.6656)	0.9478
**Liver**	**HOPE (N = 10)**	**Control** **(N** = **30)**	**Odds ratio/Effect size** **(CI interval)**	**p value**
**PNF**, **n (%)**	**0 (0%)**	**2 (6.6%)**	**0.9633 OR (0.3377–2.5797)**	**0.8942**
***EAD***, ***n (%)***	***0 (0%)***	***7 (23.3%)***	0.7667 OR (0.2734–2.1501)	0.6135
***Peak AST within 7 days (U/L)***, ***median (range)***	***344.5 (166–1132)***	***637 (124–2100)***	−0.82 EF (−1.55 to −0.08)	0.0060*
Peak ALT within 7 days (U/L), median (range)	330 (122–1350)	601 (114–1837)	−0.52 EF (−1.24 to 0.20)	0.1438
Bilirubin on 7 day (mg/dL), media (SD)	3.14 ± 1.54	3.62 ± 3.22	−0.16 EF (−0.88 to 0.55)	0.5386
***INR on 7 day***, ***median (range)***	***1.17 (1.08–1.46)***	***1.24 (1.02–1.64)***	−0.60 EF (−1.32 to 0.13)	0.0434*
*Hospital stay (days), median (range)*	*11.5 (7–29)*	*12.5 (7–109)*	−0.28 EF (−1.00 to 0.44	0.2350
***1-year graft survival***, ***n (%)***	***10 (100%)***	***27 (90%)***	0.9000 OR (0.3248–2.4937)	0.8394
***1-year recipient survival***, ***n (%)***	***10 (100%)***	***27 (90%)***	0.9000 OR (0.3248–2.4937)	0.8394

eGFR, Estimated Glomerular Filtration Rate; INR, International normalized ratio; PNF = primary non function; DGF = delay graft function; EAD = early allograft dysfunction; AST = aspartate transaminase; ALT = alanine transaminase.

**Figure 1:**
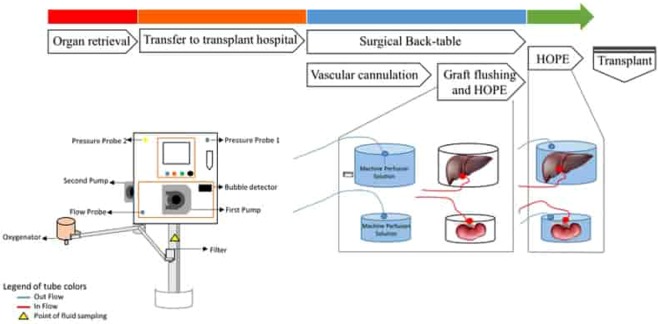
Peak AST within 7 days of the liver SCS and HOPE groups. AST, aspartate aminotransferase; SCS, static cold storage; HOPE, hypothermic oxygenated perfusion.

L-HOPE group had 0% of EAD versus 23.3% of L-SCS group. As result of this data, the peak of aspartate aminotransferase within 7-days post-LT decreased over 50%: the median value of AST was 637 (124–2100) U/L in L-SCS group *vs*. 344 (166–1032) U/L in L-HOPE group, p = 0.006 (Fig. 1). Also, alanine aminotransferase, bilirubin and INR levels were lower in L-HOPE recipients than in the L-SCS group.

Liver graft assessment following transplantation (L-GrAFT) risk score decreased significantly in the L-HOPE group, with a L-GrAFT risk score percentage of 18.82 ± 7.10%, compared to 27.03 ± 11.36% in L-SCS group (p = 0.047).

Looking at the kidney transplant subset, the study population showed a trend to a better outcome, with only 20% of DGF compared to 40% in the control group. The only two DGF events in the HOPE-K group took place when the perfusion time for single KT was lower than 2 hours; the remaining 8 cases in the HOPE-K group had no DGF events. Comparing K-HOPE patients receiving a graft which underwent perfusion treatment for more than 2 hours to grafts with shorter perfusion time, the rate of DGF was statistically significant (0% vs. 40%, p = 0.04). Overall, early post-operative complications according to the Clavien-Dindo classification were comparable among the study groups, without reaching any statistical significance (see Supplementary Materials Tables 3, 4).

### Biliary/ureteral strictures and organ rejection

Biliary and ureteral strictures were monitored for 1 year of follow-up after transplant, without observing differences in HOPE recipients *vs*. SCS group [L-HOPE group = 1/10 (10%) versus L-SCS group = 3/30 (10%); K-HOPE group = 1/10 (10%) versus K-SCS group = 2/30 (6.6%)]. Likewise, the incidence of acute rejection was similar between study and control groups [L-HOPE group = 1/10 (10%) versus L-SCS group = 4/30 (13.3%); K-HOPE group = 1/10 (10%) versus K-SCS group = 2/30 (6.6%)].

### Hospitalization, graft and patient survival

No significative differences were reported in term of median hospital stay for LT (11 days HOPE *vs*. 12 days SCS) and KT (17 days HOPE vs. 24 days SCS). All grafts and patients in the HOPE groups survived at 1-year post-transplant, with no significative differences when compared to control groups. In the L-SCS group graft survival was 90% due to two PNF events and one case of multiorgan failure secondary to sepsis; moreover, patients’ survival was 90%: one death for infectious complication and two for recurrence of liver disease. In the K-SCS group, graft survival was 93.3% due to two graft failure cases, one due to renal vein thrombosis in single renal transplant and one due to multiorgan failure following bacterial sepsis in dual renal transplant; patient survival was 96.6% due to one event of death for infectious complication (Table 4).

### NGAL in perfusate correlates with better renal transplant outcome

**Figure 2:**
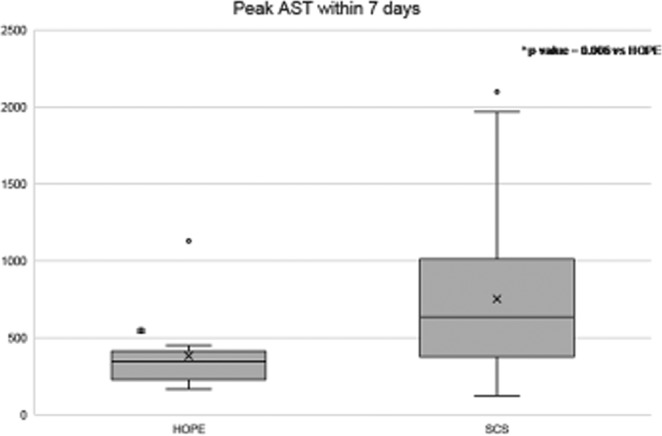
NGAL in perfusate positively correlates with eGFR at 1^st^, 3^rd^ and 6^th^ month after kidney transplant.

We splitted HOPE-K recipients in two different subsets, based on NGAL median value detected at T1 (19 pg/mL) and we monitored eGFR of HOPE-K group at 1^st^, 3^rd^ and 6^th^ month after kidney transplantation: patients with perfusate NGAL > 19 pg/mL reported significantly higher eGFR at each time point (Fig. 2). Non statistically significant correlations between other markers of acute kidney injury and eGFR at 1^st^, 3^rd^ and 6^th^ month after kidney transplantation are shown in Supplementary Materials Fig. 1.

In the liver perfusate (HOPE-L), pGST, Albumin and Beta2-microglobulin were detected with increased values at T2 when compared to T0 (Supplementary Materials Table 5), but no statistically significant linear correlations are evidenced when compared with AST, ALT and total bilirubin (at 1^st^, 3^rd^ and 6^th^ month after liver transplantation), or CIT and time of perfusion (Table 3).

We also measured NGAL, Calbindin, Clusterin and Osteopontin in the liver perfusate: we reported different values in T1 compared to T0 (Supplementary Materials Table 5) with non statistically significant linear correlations when compared with AST, ALT and total bilirubin (at 1^st^, 3^rd^ and 6^th^ month after liver transplantation), or CIT and time of perfusion (Table 3).

Results are further detailed in Supplementary Materials.

## Discussion

The present prospective pilot non-randomized clinical study suggests how hypothermic oxygenated machine perfusion results effective and safe in both liver and kidney transplantation. While administration of oxygen through machine perfusion for liver preservation was largely investigated in a previous clinical study^20^, few reports described HOPE in KT^21^. Since 1964, several *in vivo* and preclinical studies report the oxygen use in hyperbaric and normobaric conditions to perfuse renal grafts^22,23^. Here, we report the first clinical trial of HOPE in kidney transplant, reporting safety and efficacy in reducing ischemic preservation injuries.

The use of the same perfusion device for liver and kidney grafts results as one of the most innovative finding of the present study compared to other previous studies^11,13,14,24^: organ perfusion with this machine is easily performed and it may be initiated at the time of surgical graft preparation during the “back-table” procedures, avoiding unnecessary CIT extension.

An increasing number of researches suggest how the beginning of hypothermic oxygenated perfusion soon after graft retrieval promotes the reduction of waste products, such as succinates, that usually accumulate in the graft during static storage, and increasing the restoring of mitochondrial function^25,26^.

Our protocol begins with a 30–40 minutes graft washing during organ preparation, with the aim to remove waste products commonly released and accumulated during ischemia time. Furthermore, we equipped our organ perfusion system with an adsorbing hemofilter able to remove cytokines and to avoid fat embolism. After the washing phase, oxygenation of the preservation solution begins, with recirculation of the same fluid. We believe this strategy may replace the necessity to immediately start graft perfusion at the end of the retrieval, if CIT is maintained low, as in our study. Metabolites quantification in the perfusates during the flushing phase, compared to the usual perfusion time, may demonstrate the benefit of the washing strategy prior to recirculation of HOPE. The limit of our study is due to the lack of these data.

The missing of additional perfusates at regular times during perfusion, may represent a further limitation: a regular analysis of perfusates could be helpful to better understand organ metabolism during perfusion.

In the present study no adverse events were reported as a consequence of perfusion treatment and the outcome was excellent, with no occurrence of graft dysfunction in HOPE-L despite advanced donor age. No graft failure was registered in HOPE-K, and only two cases had DGF. Moreover, recipients in HOPE-K with DGF had undergone perfusion treatment for less than two hours, as compared to non-DGF patients which actually underwent perfusion treatment for more than 2 hours. Therefore, based on our experience, we suggest that kidney perfusion should last at least two hours; this treatment time could be possibly longer, as suggested by a pre-clinical study^27^.

This clinical series – to the best of our knowledge – is the first report of HOPE in ECD kidney transplantation; therefore, there are no data about renal preservation issues to compare our results with. Similarly to HOPE-L which is performed for at least one-hour, HOPE for renal grafts should last at least two hours, but future randomized trials are needed to clarify this hypothesis^11^.

Many studies, even from our center, showed that hypothermic oxygenation during organ perfusion is able to restore ATP levels in liver and kidney grafts, improving the functional recovery after transplantation^19,28,29^. Our clinical results showed a better liver function with HOPE compared to SCS in the setting of marginal donation, without reported EAD events and lower hepatocellular enzyme release. A difference in terms of graft function was more evident in the liver transplant subset, where we observed a 50% AST peak reduction in the liver HOPE group compared to the control group. This result is similar to that reported by Nasralla and colleagues in a controlled prospective randomized trial on liver normothermic perfusion following organ retrieval^30^.

Furthermore, in the liver subset, post-operative liver function was improved in the liver HOPE group according to the L-GrAFT risk score.

In addition to this positive clinical data, other relevant aspects of HOPE should be considered: first of all, the procedure proposed is simple in terms of organization and management; in particular, in our experience, a specialist perfusion technician was not required, and we had no adverse events related to the procedure. Secondly, the perfusion machine is cost-effective and less expensive compared to the subnormothermic or normothermic techniques. HOPE is performed requiring only a sterile perfusion line kit, including an oxygenator; on the other hand, subnormothermia or normothermia conditions require packed red blood cells or haemoglobin-based oxygen carriers, various perfusion solutions (i.e. Ringer’s solution), nutrient elements including multivitamins, sodium bicarbonate, heparin, antibiotics, prostacyclin, nutrient solution and creatinine. A further positive aspect of HOPE management, is the effectiveness of the strategy proposed despite advanced donor age in liver but also in kidney transplantation, with no graft loss due to non-function or dysfunction reported.

In this manuscript, we report only 1-year of clinical outcomes follow-up, including organ function, graft and patient survival; nevertheless the lower L-GrAFT risk score reported in the liver study group may suggest a better graft survival with a prolonged follow-up.

Recently, 5-year outcomes in donors after circulatory death (DCD) human livers preserved with HOPE were reported similar to DBD liver grafts and superior to the DCD liver grafts preserved with SCS^31^. Long-term outcomes of machine perfusion in renal storage was reported only in the setting of hypothermic machine perfusion without oxygen. However, graft and patient survival were superior in the machine perfusion groups compared to the SCS groups, with the equalization of usage costs achieved at the 16^th^ month post-transplant^32^. In our opinion, the analyses of the long-term results of this clinical series could be of interest, as well as other similar research studies with higher sample size.

Simplicity, outcome benefits for liver and kidney transplantation and low-costs equipment are the winning points of our HOPE system. The safety of our new machine perfusion system was confirmed by the absence of adverse events and by the observation that liver hypothermic oxygenation could be effective to improve graft function, similarly to other reported series in the setting of normothermic blood perfusion^30,33^.

We also measured different markers in perfusates at the beginning and the end of perfusion procedure. Regarding KT, NGAL resulted a positive predictive biomarker of renal outcomes at 1^st^, 3^rd^ and 6^th^ month of follow up: despite NGAL is largely known as a marker for acute and chronic renal injuries^34–36^, this result partially correlates with our previous findings, suggesting the role of NGAL as an immunomodulatory molecule, able to promote immune tolerance through regulatory T cells (Treg)^37,38^ therefore favoring better clinical outcomes.

In LT, significative differences of all perfusate markers were reported when measured at the end of perfusion if compared with the beginning. Recently Dutkosky *et al*.^39^ demonstrated that mitochondrial flavin levels in perfusates during hypothermic oxygenated perfusion might be a marker of better liver graft function. Our preliminary results confirm the relevance of markers in the perfusate and the fact that perfusate analysis is a further tool to predict clinical grafts outcomes. We are currently exploring a new field where further studies involving a larger sample size and a more comprehensive panel of biomarkers are needed, with the aim to detect optimal targetable markers to correlate with graft function or to confirm the results of Zurich center.

Summarizing, we consider our innovative system as a functional tool to allow marginal grafts transplantability, with the aim to reduce utilization avoidance of grafts which are actually discarded^19,40,41^. Moreover, the analysis of the perfusate may help to evaluate organ viability even in hypothermic conditions, and the infusion of therapeutic agents – e.g. nutrition, mesenchymal stromal cells (MSC) and extracellular vesicles releasing by MSC, anti-inflammatory and anti-apoptotic additives – might be a simple implement of the perfusion system in use^42–45^.

Future trials exploring this field must confirm not only safety and benefits, but also cost effectiveness, as some perfusion strategies are still associated with exorbitant costs.

In conclusion, HOPE for ECD-DBD grafts is safe and effective in reducing preservation injury and in improving graft function and outcomes for kidney and liver transplantation. Future randomized clinical studies with a representative sample size are needed to confirm our results and hypothesis.

## Methods

### Study design

This national, single-center, prospective interventional arm with a retrospective case-controlled arm, pilot non-randomized clinical study was performed at the General Surgery and Transplant Unit of University of Bologna Sant’Orsola - Malpighi Hospital in accordance to the Helsinki Declaration. Ethical approval was obtained by local committee of University of Bologna Sant’Orsola - Malpighi Hospital in June 2016. The enrolment process of the interventional study arm was planned considering one procedure per month (liver or kidney perfusion before transplantation) following the inclusion criteria in the period from October 2016 to December 2017. The study was meant to evaluate the safety of a new machine perfusion system, and therefore we decided to monitor adverse events after each procedure for the first 30 post-operative days before enrolling other new cases. During the study period we performed 116 LTs, 60 of which met the inclusion criteria and 10 of which were included in the study according to the aforementioned safety protocol. Similarly we recluted 60 KTs, which met the inclusion criteria, and among those 10 were included according to the protocol. The study underwent clinical trial registration (ClinicalTrials.gov ID: NCT03031067; 01/25/2017). The organ allocation process followed our center algorithm^6,8,46,47^. Each patient included in the study group was transplanted with ECD graft, liver or kidney, restored in the pre-implantation phase by HOPE. Patients’ outcomes (HOPE-L and HOPE-K groups) were compared to the ones of matched control groups composed by liver and kidney recipients transplanted from January 2004 to September 2017 in the same transplant center, whose organs were preserved by SCS (SCS-L and SCS-K groups respectively).

Demographical, clinical, machine perfusion and histological data collection was performed prospectively for the study groups and retrospectively for the control groups.

### Matching study and control cases

Study cases of HOPE-L and HOPE-K groups were matched 1:3 to control cases of SCS-L and SCS-K groups; the pairing was performed anonymously using different clinical features about each donor and recipient involved.

HOPE-L and SCS-L groups were matched for donor and recipient age, CIT, model of end-stage liver disease (MELD) score, previous abdominal surgery and portal thrombosis.

HOPE-K and SCS-K groups were matched for donor and recipient age, (CIT), Karpinski’s score, dialyses type (peritoneal or hemodialysis) and dialyses time.

### Recipient and donor selection

ECD-DBDs aged over 18 years were considered eligible for study inclusion after acquisition of informed consent by the family. Definition of ECD was applied according to the UNOS criteria for kidney transplant (donors aged ≥60 years or aged 50–59 years with two or more other risk factors such as cerebrovascular accident, hypertension and serum creatinine >1.5 mg/dL) and for liver transplant (donors with hemodynamic deterioration, age >65 years, BMI > 30 kg/m^2^, bilirubin >3 mg/dL, aspartate aminotransferase (AST) o alanine aminotransferase (ALT) above three times the upper reference threshold, sodium >165 mmol/l, days on intensive care unit (ICU) > 7, steatosis >40%, CIT > 14 hours^48,49^).

Recipients aged over 18 years on liver/kidney waiting list to University of Bologna Sant’Orsola - Malpighi Hospital with favorable donor match were selected and included in the trial after acceptation of the informed consensus.

Combined organ transplant, donors or recipients with vascular anomalies, urgency transplants and re-transplantations were excluded from the study enrollment.

### Organ retrieval and transfer

Livers/kidneys were harvested in a standard fashion and according to our policy for ECD^5,50–52^. At the end of the retrieval procedures, the organs were stored in cooling sterile bag with new SCS solution and transported to the transplant hospital, where we started the perfusion protocol in the study group.

### Liver graft histopathological analysis

In order to verify the homogeneity between HOPE-L and SCS-L groups, as well as between HOPE-K and SCS-K groups, the graft histopathology was revised by two different pathologists in all cases.

### Hypothermic oxygenated perfusion

After notification to the Health National System for the clinical use in organ perfusion, an innovative machine perfusion developed and described in a previous experimental study^19^ was used to perform *ex-vivo* hypothermic oxygenated perfusion.

This machine perfusion is composed by two pump units that produce a continuous and pulsatile flow to the vein or artery respectively, which is set at high values for liver perfusion (median value over 100 mL/min) and at lower values for kidney perfusion (median value minor to 60 mL/min).

Before HOPE set-up, the inner and outer sterile flow tubes were connected to the medical devices and the priming phase was started. Belzer University of Wisconsin machine perfusion solution was used for priming and organ perfusion steps. At the begin of surgical back-table, portal vein or renal artery were cannulated with special cannulas for organ perfusion of different sizes (8–12 F for kidney and 18–24 F for liver). Each organ was connected to the perfusion device through the attachment of the vascular cannula to the inner perfusion tubes, already assembled to the machine. HOPE was performed at 4 °C, with a portal vein pressure of 5 mmHg or renal artery pressure of 25 mmHg and an oxygen partial pressure (paO_2_) of 600–750 mmHg. Oxygen is provided to the fluid perfusion through the addition of a membrane oxygenator to the perfusion circuit. Flow, pressure and temperature values were monitored frequently by the organ perfusion team involved and registered on USB memory support. Perfusate gas analysis were accomplished every 15 minutes on effluent perfusate samples to measure paO_2_, pH, and lactate production.

Sterility of perfusion treatment under study was tested performing microbial cultures on perfusion fluid before (T0) and after HOPE (T1).

Hypothermic oxygenated perfusion was performed in two steps: the first one was the flushing of the organ with hypothermic oxygenated solution for a duration of 30–40 minutes, and during the second step the organs were moved in a different box to permit recirculation of the perfusate until implantation.

**Figure 3:**
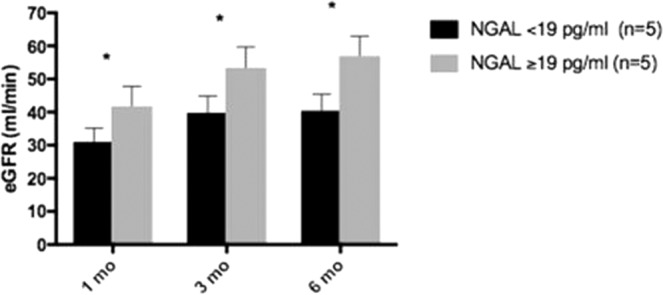
Algorithm treatment in the study group: organ retrieval, hospital transfer, back-table with HOPE and flushing of graft and after HOPE until transplantation.

Graft flushing started at the beginning of the back-table phase, thus without increasing the duration of the standard surgical steps. Flushing was performed with hypothermic oxygenated solution and after 30–40 minutes of graft flushing, the organs were moved in the another organ container to permit recirculation of the oxygenated perfusate. The algorithm treatment is reported in Fig. 3.

### Transplantation

According to the conventional procedures, surgical team performed kidney and liver transplants. Renal transplants were accomplished into iliac fossa anastomosing the artery to the external/common/internal iliac arteries, the vein to the external/common iliac veins and the ureter to the bladder over a single stent. Orthotopic liver transplantation was performed with “piggyback” technique preserving the inferior vena cava; epato-choledocal anastomoses were performed over T-tube.

### Short and long-term post-operative course

Post-transplant medical care during the hospital stay, including antithrombotic and antimicrobial/antifungal prophylaxis, was managed applying the standard protocols^53,54^. According to transplant guidelines, immunosuppressive state of recipients was preserved administering induction therapy for KT, tacrolimus (optimal levels: 3–12 ng/mL) with or without mycophenolate as either mycophenolatemofetil or mycophenolate sodium steroid dose decreasing. Long-term follow-up was carried out according to the local protocols at 1-, 3-, 6- and -12 post-transplant months, in a single-center without loss of patients at follow-up.

### Transplant outcome

Graft survival as time, incidence of PNF and graft function as percentage of Liver Graft Assessment Following Transplantation (L-GrAFT) risk score or incidence of EAD for LT or DGF for KT were the measure tools to establish the HOPE effects on ECD-DBD donation in LT and KT. PNF was defined as organ failure ending to re-transplant for LT or constant dialyses recovery/transplantectomy for KT. EAD was defined if one or more of these lab values appeared: bilirubin>10 mg/dL on post-operative day 7; INR > 1.6 on post-operative day 7; aminotransferase level (ALT or AST) > 2000 IU/mL within the first 7 post-operative days^55^. LGrAFT risk score was calculated on 1–10 post-LT days of AST, bilirubin, platelet counts and maximum INR values^56^. DGF was defined as the needing of dialyses treatment within the first week after the transplant; the effect on the outcome is expressed as an absolute number outcome^57^. Other follow-up data under study were: functional lab test values at 1-, 3-, 6- and -12 post-transplant months, incidence of biliary/ureteral fistula, incidence of graft rejection and the number of hospital readmission within 1 year from transplant.

### Perfusate analyses

5 mL of perfusate were collected at the beginning (T0) and at the end (T1) of perfusion from the perfusion fluid in the organ container and centrifuged at 2500 rpm for 15 min to remove cellular debris. The supernatant was transferred to 1-mL Coming cryogenic vials immediately after centrifugation and stored at −80’C for subsequent analysis. Samples were thawed at room temperature prior to use for analyses. The concentrations of early biomarkers of renal injury (Neutrophil Gelatinase-Associated Lipocalin-NGAL, Osteopontin, Beta2-microglobulin, Clusterin, Cystatin-C, Calbindin, Monocyte Chemoattractant Protein-1-MCP) and specific markers of liver functions (Glutathione S-transferases phosphorylate-pGST, ALbumin, Beta2-microglobulin) were measured using Luminex® xMAP® Technology. We created our own multiplex panel by combining multiple simplex kits: Human CustemProcarta Plex-19 plex (Invitrogen by Thermo Fisher Scientific, Cat. No.PPX-19-MXRWE2G) and Human High Sensitivity T Cell (Merck Millipore, Cat. No. HSTCMAG-28SK). The methodological details including assay protocol, standards and sensitivity are available at the manufacturer’s website, http://www.thermofisher.com and http://www.merckmillipore.com. All samples were measured undiluted and in doublets. The chemo/cytokine standards were assayed in the same manner as patient samples. The data were collected using a xPONENT® software (Luminex, Austin, TX, USA).

### Study end points

The primary end point were the safety in the interventional arm and differences in terms of graft function between the two study groups. Graft function was measured considering the EAD for the liver and the DGF for the kidney. Liver function was also evaluated considering the L-GrAFT risk core and the peak of aspartate aminotransferase within 7-days post-LT.

The secondary en points included: graft and patient survival at one-year, post-operative complications according to Clavien-Dindo classification and hospital stay.

### Statistical analyses

Continuous data are reported as mean ± SD or median (range) depending on their distribution and compared by means of parametric (ANOVA, T test) or non-parametric (Mann-Whitney U test) analyses. Categorical data are presented as percentage values and compared using a Chi-squared test. Kaplan-Meier estimators were applied to perform the survival analysis. A p value <0.05 was considered as statistically significant.

Further details are provided in the Supplementary Material.

### Organs/tissues procured

NO organs/tissues were procured from prisoners. Organs/tissues were procured from various institutions. Ospedale Maggiore – Bologna (BO), Ospedale Maggiore – Parma (PR), Nuovo Ospedale S. Agostino Estense - Baggiovara (MO), S.Maria Nuova - Reggio nell’Emilia (RE), Ospedale M. Bufalini – Cesena (FC), Ospedale Infermi - Rimini (RN), Arcispedale S. Anna – Ferrara (FE), Policlinico- Modena, S. Orsola Malpighi - Bologna (BO), Ospedale Civile- Piacenza (PC), Ospedale Santa Maria della Scaletta - Imola (BO), Ospedale Bellaria - Bologna (BO), Ospedale Santa Maria delle Croci - Ravenna (RA), Ospedale degli Infermi-Faenza (RA), Ospedale Morgagni Pierantoni - Forlì (FC).

### Trial Registration

Protocol IDPIO-700; ClinicalTrials.gov ID: NCT03031067; Date of registration 01/25/2017.

## Supplementary information

Supplementary materials.

## Data Availability

Data sharing is not applicable to this article as no datasets were generated or analysed during the current study.

## References

[1] Hart A (2017). OPTN/SRTR 2015 Annual Data Report: kidney. Am J Transplant..

[2] Mathur AK (2014). Variation in access to the liver transplant waiting list in the United States. Transplantation..

[3] Hart A (2018). OPTN/SRTR 2016 Annual Data Report: Kidney. Am J Transplant..

[4] Kim WR (2018). OPTN/SRTR 2016 Annual Data Report: Liver. Am J Transplant..

[5] Cuna V (2017). Fifteen-Year Analysis of Deceased Kidney Donation: A Single Transplant Center Experience in a Region of Northern Italy. Med Sci Monit..

[6] Bertuzzo VR (2017). Actual Risk of Using Very Aged Donors for Unselected Liver Transplant Candidates: A European Single-center Experience in the MELD Era. Ann Sur..

[7] Querard AH (2016). Comparison of survival outcomes between Expanded Criteria Donor and Standard Criteria Donor kidney transplant recipients: a systematic review and meta-analysis. Transpl Int..

[8] Beal EW (2017). High Center Volume Does Not Mitigate Risk Associated with Using High Donor Risk Organs in Liver Transplantation. Dig Dis Sci..

[9] Głyda M, Włodarczyk Z, Czapiewski W (2012). Results of renal transplantation from expanded criteria deceased donors - a single-center experience. Ann Transplant..

[10] Jochmans I (2016). Past, Presente and Future of Dynamic Kidney and Liver Preservation and Resuscitation. Am J Trasnplant..

[11] Dutkowski P (2015). First Comparison of Hypothermic Oxygenated PErfusion Versus Static Cold Storage of Human Donation After Cardiac Death Liver Transplants: An International-matched Case Analysis. Ann Surg..

[12] Kron P (2016). Short, Cool, and Well Oxygenated - HOPE for Kidney Transplantation in a Rodent Model. Ann Surg..

[13] Ravikumar R (2016). Liver Transplantation After *Ex Vivo* Normothermic Machine Preservation: A Phase 1 (First-in-Man) Clinical Trial. Am J Transplant..

[14] Nicholson ML, Hosgood SA (2013). Renal transplantation after *ex vivo* normothermic perfusion: the first clinical study. Am J Transplant..

[15] Hoyer DP (2014). Subnormothermic machine perfusion for preservation of porcine kidneys in a donation after circulatory death model. Transpl Int..

[16] Schopp I (2015). Controlled rewarming after hypothermia: Adding a new principle to renal preservation. Clin. Transl. Sci..

[17] Fontes P (2015). Liver preservation with machine perfusion and a newly developed cell-free oxygen carrier solution under subnormothermic conditions. Am J Transplant..

[18] Hoyer DP (2015). Controlled oxygenated rewarming of cold stored livers prior transplantation: First clinical application of a new concept. Transplantation..

[19] Ravaioli M (2018). Strategies to Restore Adenosine Triphosphate (ATP) Level After More than 20 Hours of Cold Ischemia Time in Human Marginal Kidney Grafts. Ann Transplant..

[20] Dondossola D (2019). Preliminary Experience With Hypothermic Oxygenated Machine Perfusion in an Italian Liver Transplant Center. Transplant Proc..

[21] Kataria A, Magoon S, Makkar B, Gundroo A (2019). Machine perfusion in kidney transplantation. CurrOpin Organ Transplant..

[22] Manax WG (1964). Successful 24 hour *in vitro* preservation of canine kidneys by the combined use of hyperbaric oxygenation and hypothermia. Surgery..

[23] Takasaki N (1968). [Study on renal transplantation. I. Experimental study on preservation of the kidney: method of hypothermic immersion uder normal oxygen pressure]. Hinyokika Kiyo..

[24] Kox J (2018). The Benefits of Hypothermic Machine Preservation and Short Cold Ischemia Times in Deceased Donor Kidneys. Transplantation..

[25] Schlegel A, Muller X, Dutkowski P (2018). Hypothermic Machine Preservation of the Liver: State of the Art. Curr Transplant Rep..

[26] Schlegel A, Kron P, Dutkowski P (2015). Hypothermic Oxygenated Liver Perfusion: Basic Mechanisms and Clinical Application. Curr Transplant Rep..

[27] Darius T (2018). The effect on early renal function of various dynamic preservation strategies in a preclinical pig ischemia-reperfusion autotransplant model. Am J Transplant..

[28] Buchs JB (2011). Oxygenated hypothermic pulsatile perfusion versus cold static storage for kidneys from non heart-beating donors tested by in-line ATP resynthesis to establish a strategy of preservation. Perfusion..

[29] Van Rijn R (2017). Dual hypothermic oxygenated machine perfusion in liver transplants donated after circulatory death. Br J Surg..

[30] Nasralla D (2018). A randomized trial of normothermic preservation in liver transplantation. Nature..

[31] Schlegel A (2019). Outcomes of DCD liver transplantation using organs treated by hypothermic oxygenated perfusion before implantation. J Hepatol..

[32] Wszola M (2009). Long term medical and economical benefit of machine perfusion (MP) kidney storage in comparison to cold storage (CS). Ann Transplant..

[33] Ghinolfi D (2018). Pilot, open, randomized, prospective trial for normothermic machine perfusion evaluation in liver transplantation from older donors. Liver Transpl..

[34] Mishra J (2005). Neutrophil gelatinase-associated lipocalin (NGAL) as a biomarker for acute renal injury after cardiac surgery. Lancet..

[35] Nickolas TL (2012). Forster, M.E. Sise, et al.NGAL (Lcn2) monomer is associated with tubulointerstitial damage in chronic kidney disease. Kidney Int..

[36] Cappuccilli M, Capelli I, Comai G, La Manna G (2018). Neutrophil Gelatinase-Associated Lipocalin as a Biomarker of Allograft Function After Renal Transplantation: Evaluation of the Current Status and Future Insights. Artif Organs..

[37] La Manna G (2014). Neutrophil gelatinase-associated lipocalin increases HLA-G(+)/FoxP3(+) T-regulatory cell population in an *in vitro* model of PBMC. PLoS One..

[38] Donadei C, Cappuccilli M, La Manna G (2018). An intriguing link between human leukocyte antigen G, T-regulatory cells and neutrophil gelatinase-associated lipocalin in immune tolerance induction. Cytotherapy..

[39] Muller X (2019). Novel Real-time Prediction of Liver Graft Function During Hypothermic Oxygenated Machine Perfusion Before Liver Transplantation. Ann Surg..

[40] Ravaioli M (2018). Preliminary experience of sequential use of normothermic and hypothermic oxygenated perfusion for donation after circulatory death kidney with warm ischemia time over the conventional criteria - a retrospective and observational study. Transpl Int..

[41] Ravaioli M (2017). Successful Dual Kidney Transplantation After Hypothermic Oxygenated Perfusion of Discarded Human Kidneys. Am J Case Rep..

[42] Eshmuminov D (2018). Perfusion settings and additives in liver normothermic machine perfusion with red blood cells as oxygen carrier. A systematic review of human and porcine perfusion protocols. TransplInt..

[43] Bae C (2014). The benefits of hypothermic machine perfusion are enhanced with Vasosol and α-tocopherol in rodent donation after cardiac death livers. Transplant Proc..

[44] Gregorini M (2017). Perfusion of isolated rat kidney with Mesenchymal Stromal Cells/Extracellular Vesicles prevents ischaemic injury. J Cell Mol. Med..

[45] Rigo F (2018). Extracellular Vesicles from Human Liver Stem Cells Reduce Injury in an *Ex Vivo* Normothermic Hypoxic Rat Liver Perfusion Model. Transplantation..

[46] Remuzzi G (1999). Early experience with dual kidney transplantation in adults using expanded donor criteria. Double Kidney Transplant Group (DKG). J Am Soc Nephrol..

[47] Angeletti A, Cravedi P (2019). Making Procurement Biopsies Important Again for Kidney Transplant Allocation. Nephron..

[48] Port FK (2002). Donor characteristics associated with reduced graft survival: an approach to expanding the pool of kidney donors. Transplantation..

[49] Attia M, Silva MA, Mirza DF (2008). The marginal liver donor – an update. Transplant International..

[50] Ravaioli M (2009). Liver transplantations with donors aged 60 years and above: the low liver damage strategy. Transpl Int..

[51] Di Laudo M (2017). Combined liver-dual kidney transplant: Role in expanded donors. Liver Transpl..

[52] Ravaioli M (2016). Risk Avoidance and Liver Transplantation: A Single-center Experience in a National Network. Ann Surg..

[53] Kidney Disease: Improving Global Outcomes (KDIGO) Transplant Work Group (2009). KDIGO clinical practice guideline for the care of kidney transplant recipients. Am J Transplant..

[54] Ravaioli M (2015). Immunosuppression Modifications Based on an Immune Response Assay: Results of a Randomized, Controlled Trial. Transplantation..

[55] Olthoff KM (2010). Validation of a current definition of early allograft dysfunction in liver transplant recipients and analysis of risk factors. Liver Transpl..

[56] Agopian VG (2018). Evaluation of Early Allograft Function Using the Liver Graft Assessment Following Transplantation Risk Score Model. JAMA Surg..

[57] Humar A (2002). Risk factors for slow graft function after kidney transplants: a multivariate analysis. Clin Transplant..

