# Author Correction: Hypothermic Oxygenated New Machine Perfusion System in Liver and Kidney Transplantation of Extended Criteria Donors: First Italian Clinical Trial

**DOI:** 10.1038/s41598-020-70620-y

**Published:** 2020-09-01

**Authors:** Matteo Ravaioli, Vanessa De Pace, Andrea Angeletti, Giorgia Comai, Francesco Vasuri, Maurizio Baldassarre, Lorenzo Maroni, Federica Odaldi, Guido Fallani, Paolo Caraceni, Giuliana Germinario, Chiara Donadei, Deborah Malvi, Massimo Del Gaudio, Valentina Rosa Bertuzzo, Antonio Siniscalchi, Vito Marco Ranieri, Antonietta D’Errico, Gianandrea Pasquinelli, Maria Cristina Morelli, Antonio Daniele Pinna, Matteo Cescon, Gaetano La Manna

**Affiliations:** 1 Department of General Surgery and Tran splantation, University of Bologna Sant’Orsola - Malpighi Hospital, Bologna, Italy; 2 Department of Experimental Diagnostic and Specialty Medicine, University of Bologna Sant’Orsola- Malpighi Hospital, Bologna, Italy; 3 Department of Medical and Surgical Science, University of Bologna Sant’Orsola - Malpighi Hospital, Bologna, Italy

Correction to: *Scientific Reports* 10.1038/s41598-020-62979-9, published online 08 April 2020

This Article contains an error in the order of the Figures. Figures 1, 2 and 3 were published as Figures 2, 3 and 1 respectively. The correct Figures appear below as Figures 1, 2 and 3. The Figure legends are correct.

**Figure 1:**
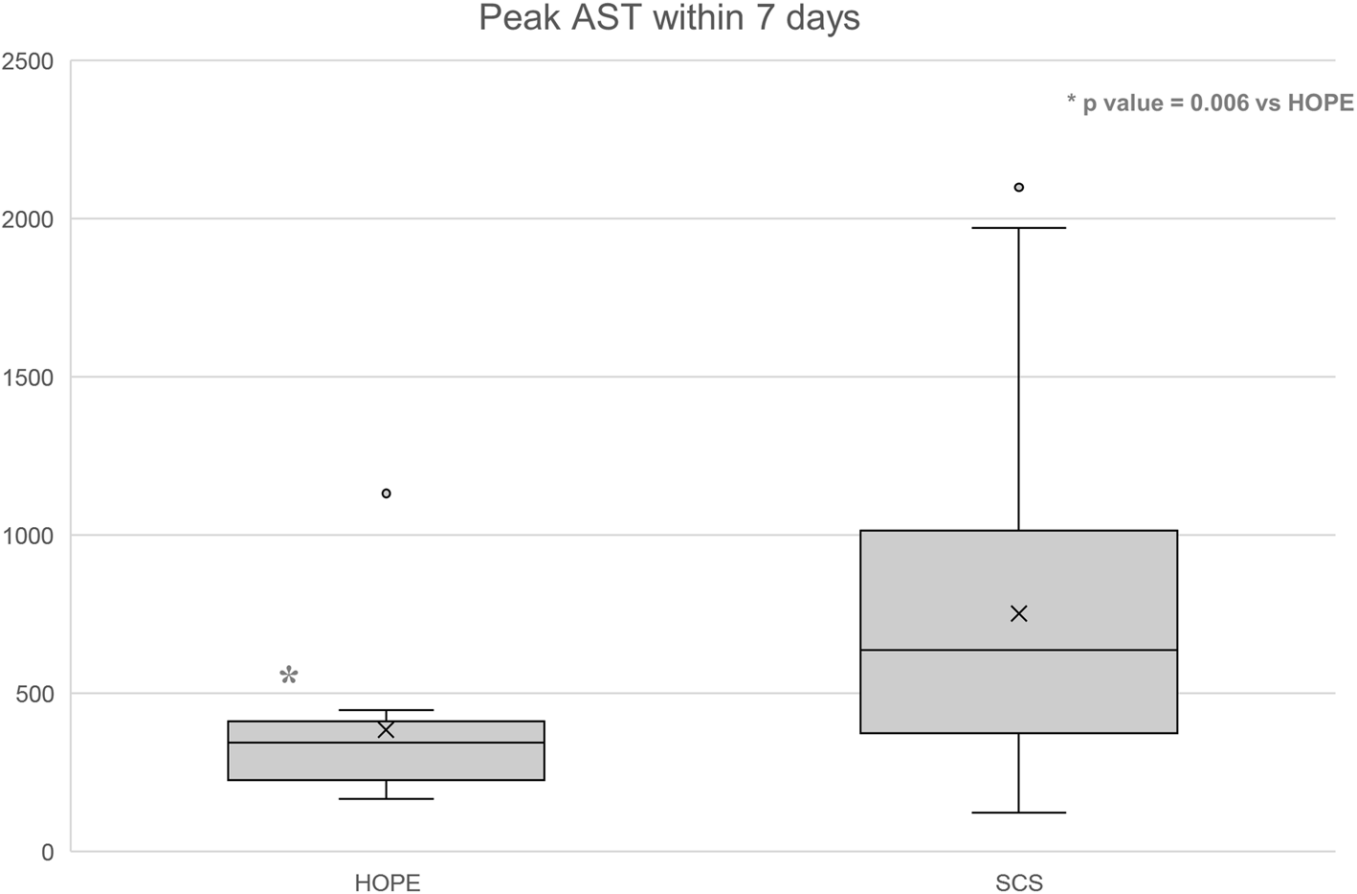
.

**Figure 2:**
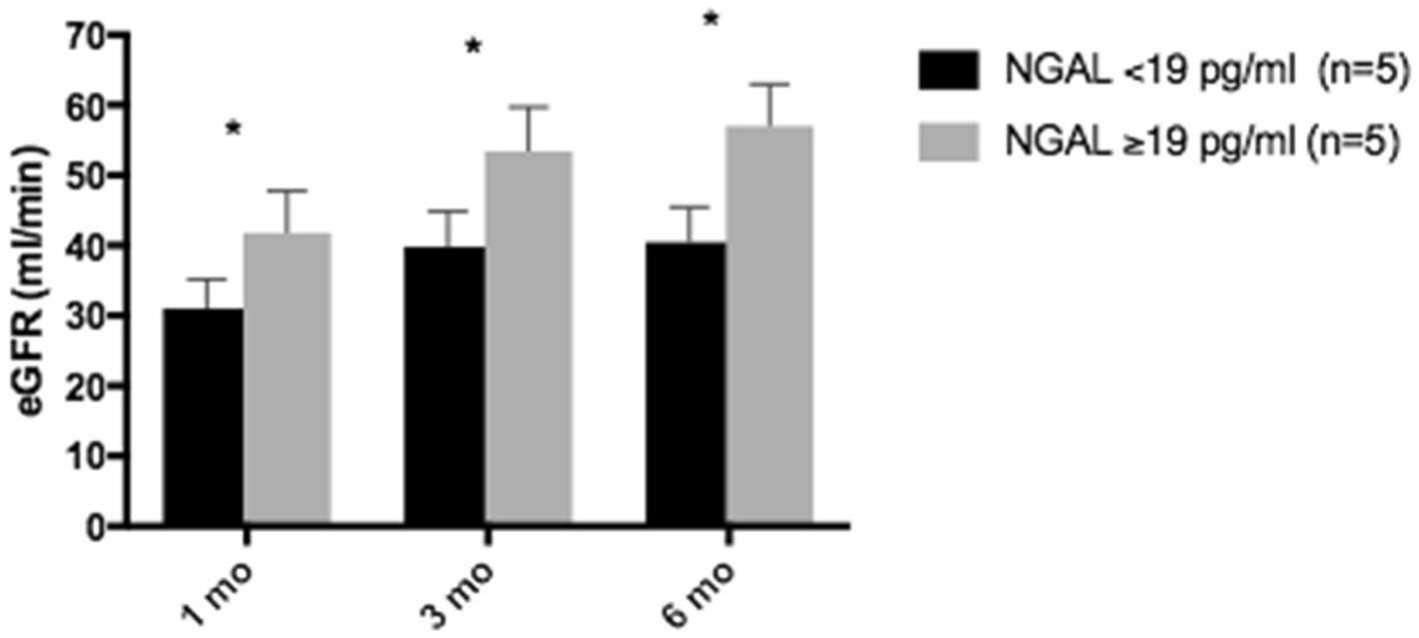
.

**Figure 3:**
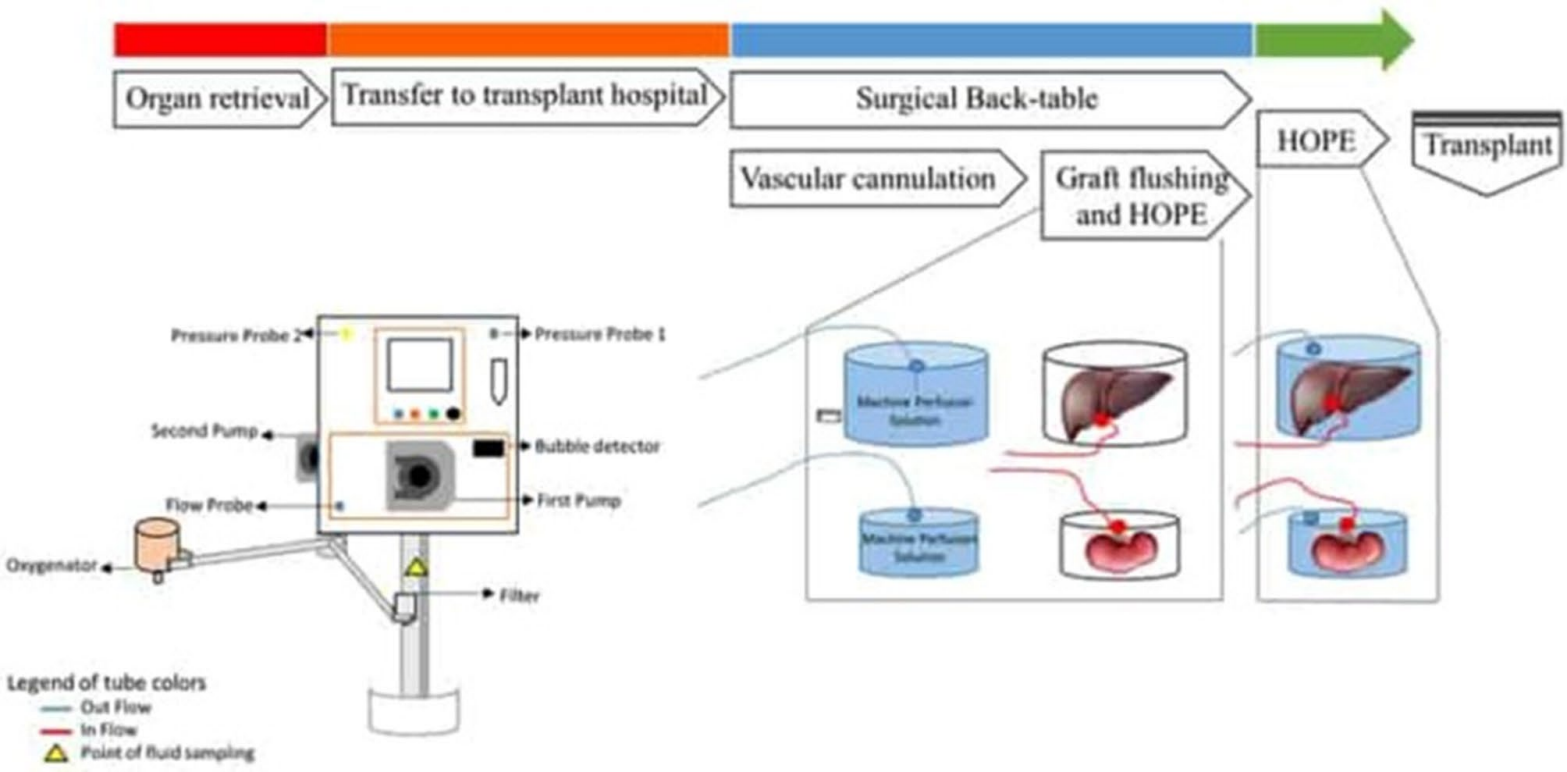
.

