# Supplementary material for: Hypothermic Oxygenated New Machine Perfusion System in Liver and Kidney Transplantation of Extended Criteria Donors:First Italian Clinical Trial
Source: Sci Rep. 2020 Apr 8;10:6063. doi: 10.1038/s41598-020-62979-9 (PMC7142134; doi:10.1038/s41598-020-62979-9)

SUPPLEMENTARY MATERIALS FOR

Hypothermic Oxygenated New Machine Perfusion System in Liver and Kidney Transplantation of Extended Criteria Donors: First Italian Clinical Trial

Matteo Ravaioli*, Vanessa De Pace, Andrea Angeletti, Giorgia Comai, Francesco Vasuri, Maurizio Baldassarre, Lorenzo Maroni, Federica Odaldi, Guido Fallani, Paolo Caraceni, Giuliana Germinario, Chiara Donadei, Deborah Malvi, Massimo Del Gaudio, Valentina Rosa Bertuzzo, Antonio Siniscalchi, Vito Marco Ranieri, Antonietta D'Errico, Gianandrea Pasquinelli, Maria Cristina Morelli, Antonio Daniele Pinna, Matteo Cescon, Gaetano La Manna.

SUPPLEMENTARY METHODS

Liver graft histopathological analysis.

Liver biopsies were sent to out Transplant Pathology Unit without any fixation media for the frozen-section analysis for graft suitability. After the frozen sections, tissue was fixed in formalin, embedded in paraffin and routinely processed. From paraffin blocks, 2-µm-thick sections were cut for permanent Haematoxylin-Eosin and Reticulin stains.

In liver grafts, according to the guidelines adopted in our Institution[1], 9 variables were separately evaluated:

- Percentage of macrovesicular steatosis;
- Percentage of microvesicular steatosis;
- Portal fibrosis according to Metavir[2];
- Amount of portal inflammatory infiltrate and fibrosis according to Ishak[3];
- Amount of lobular necrosis/inflammatory infiltrate according to Ishak;
- Arteriolar myointimal thickening, scored as absent, mild, moderate and severe;

- Biliocyte regressive changes, scored as absent, focal or diffuse;
- Presence of ductular reaction/neoduttulogenesis;
- Presence of cholestasis.

Kidney graft histopathological analysis.

Kidney biopsies were sent to out Transplant Pathology Unit in Serra solution for the histological analysis for graft suitability. Tissue was rapidly processed in microwave oven. From paraffin blocks, 2-µm-thick sections were cut for permanent Haematoxylin-Eosin, Masson's Trichrome and Periodic Acid Schiff stains. Thirteen variables were separately evaluated:

- Glomerulosclerosis, according to Karpinski[4];
- Tubular atrophy, according to Karpiski;
- Interstitial fibrosis, according to Karpiski;
- Vascular damage, according to Karpiski;
- Total Karpinski's score[5];
- Glomerular ischemic changes, scored as absent, focal or diffuse;
- Mesangial matrix thickening, scored as absent, mild or moderate;
- Presence of thrombotic microangiopathy[6];
- Acute tubular necrosis, scored as absent, focal or diffuse[23];
- Isometric vacuolization of tubulocytes, scored as absent, focal or diffuse;
- Presence of intraluminal calcifications' tubuli;
- Prevalence of sclerotic or hyaline changes in arterial/arteriolar walls;
- Presence of interstitial inflammatory infiltrate.

Hypothermic oxygenated perfusion

Organ perfusion has been started during the graft preparation at the surgical back-table in flushing at controlled pressure and flow (20 ml/min) and with new perfusion solution. Concluded the surgical preparation after 30-40 minutes, HOPE has been continued normally at controlled pressure only.

REFERENCES

1. Ravaioli M, Grazi GL, Cescon M, et al. Liver transplantations with donors aged 60 years and above: the low liver damage strategy. *Transpl Int.* 2009; 22:423-33.
2. Di Laudo M, Ravaioli M, La Manna G, et al. Combined liver-dual kidney transplant: Role in expanded donors. *Liver Transpl. 2017;* 23:28-34.
3. Ravaioli M, Grande G, Di Gioia P, et al. Risk Avoidance and Liver Transplantation: A Single-center Experience in a National Network. *Ann Surg. 2016;* 264: 778-786.
4. Fiorentino M, Vasuri F, Ravaioli M, et al. Predictive value of frozen-section analysis in the histological assessment of steatosis before liver transplantation. *Liver Transpl. 2009;*15:1821-1825.
5. The French METAVIR Cooperative Study Group. Intra observer and inter observer variations in liver biopsy interpretation in patients with chronic hepatitis C. *Hepatology.* 1994; 20:15-20.
6. Ishak K, Baptista A, Bianchi L, et al. Histological grading and staging of chronic hepatitis. *J. Hepatol.* 1995; 22:696–699.

Supplementary Table S1 | Frequencies and percentages of the 9 histopathological variables evaluated in HOPE-L and SCS-L. No differences were found between the two groups.

	**Sperimental Group**	**Control group**	**P value**
	**HOPE**	**SCS**	
Liver Transplantation	*N = 10*	*N = 30*	
Fibrosis (>2 Ishak)	2 (20%)	6 (20%)	0.524
Moderate portal inflammation	1 (11%)	5 (17%)	0.408
Lobular necrosis	2 (20%)	4 (13%)	0.352
Moderate/severe myointimal thickening	5 (50%)	14 (47%)	0.682
Biliocyte regressive changes	6 (60%)	19 (63%)	0.458
Ductular reaction	1 (10%)	6 (20%)	0.483
Cholestasis	2 (20%)	5 (17%)	0.527
Mean macrovesicular steatosis	3.1 ± 2.4	3.9±5.2	0.677
Mean microvesicular steatosis	8.11 ± 8.4	8.9±8.1	0.921

Supplementary Table S2 | Frequencies and percentages of the 13 histopathological variables evaluated in HOPE-K and SCS-K. No differences were found between the two groups.

	**Sperimental Group**	**Control group**	**P value**
	**HOPE**	**SCS**	
Kidney Transplantation	*N = 11*	*N = 30*	
Glomerulosclerosis (Karpinski's 1-2)	8 (73%)	11 (41%)	0.085
Tubular atrophy (Karpinski's 1-2)	6 (54%)	23 (77%)	0.317
Interstitial fibrosis (Karpinski's 1-2)	6 (64%)	21 (70%)	0.280
Vascular damage (Karpinski's 2-3)	4 (36%)	5 (17%)	0.639
Mean Karpinski's score	3.6±2.0	3.4±1.3	0.639
Ischemic glomeruli	6 (55%)	8 (27%)	0.108
Mesangial matrix thickening	8 (73%)	16 (53%)	0.497
Glomerular microangiopathy	0 (0%)	3 (10%)	0.296
Acute tubular necrosis (diffuse)	4 (36%)	5 (17%)	0.271
Vascular changes:- sclerosis	8 (73%)	21 (70%)	0.591
- hyalinosis	3 (28%)	9 (30%)	
Isometric vacuolization of tubuli	6 (64%)	9 (30%)	0.060
Calcifications of tubuli	1 (9%)	0 (0%)	0.324
Interstitial inflammatory infiltrate	3 (28%)	5 (17%)	0.404

Supplementary Table S3 | Post-operative complication according to Clavien Dindo grade classification

Kidney-Clavien Dindo grade	*HOPE (N = 10)*	*SCS (N = 30)*
I	0/10 (0%)	2/30 (6.7%)
II	1/10 (10%)	5/30 (16.7%)
IIIa	0/10 (0%)	1/30 (3.3%)
IIIb	1/10 (10%)	1/30 (3.3%)
IVa	2/10 (20%)	12/30 (40%)
IVb	0/10 (0%)	1/30 (3.3%)
V	0/10 (0%)	0/30 (0%)
Total	4/10 (40%)	22/30 (73.3%)
Liver–Clavien Dindo grade	*HOPE (N = 10)*	*SCS (N = 30)*
I	1/10 (10%)	4/30 (13.3%)
II	5/10 (50%)	10/30 (33.3%)
IIIa	1/10 (10%)	2/30 (6.7%)
IIIb	0/10 (0%)	1/30 (3.3%)
IVa	0/10 (0%)	3/30 (10%)
IVb	0/10 (0%)	0/30 (0%)
V	0/10 (0%)	1/30 (3.3%)
Total	7/10 (70%)	21/30 (70%)

Supplementary Table S4 | Description of post-transplant complications

Kidney complication	*HOPE*	*SCS*
Renal Replacement Therapy	2/10	12/30
Anemia	2/10	3/30
Ureteral stenosis	1/10	3/30
Infection	2/10	6/30
Acute rejection	1/10	2/30
Atrial fibrillation	0/10	3/30
Venous thrombosis	0/10	1/30
Bleeding	0/10	1/30
Acute pancreatitis	0/10	1/30
Dysuria	0/10	1/30
Liver complication	*HOPE (N=10)*	*SCS (N=30)*
Psychomotor agitation	2/10	1/30
Infection	4/10	4/30
Ascites	1/10	6/30
Biliary stenosis	1/10	0/30
Biliary leak	0/10	1/30
Anemia	1/10	1/30
Pleural effusion	0/10	3/30
Atrial fibrillation	0/10	1/30
Bleeding	0/10	1/30
Acute rejection	0/10	3/30
Renal insufficiency	0/10	1/30
Arterial thrombosis	0/10	1/30
Cholestasis	0/10	1/30

Supplementary Table S5 | Markers in Liver Perfusate.

Values are expressed as pg/ml (GST P is expressed as MFI) ± Standard Deviation.

T0 values were detected in 3 different samples Belzer solutions before starting perfusion

Markers	T0	T1	*p*
pGST	2043	42,544±67,400	*0.002*
beta2-microglobulin	3	28.6±22.3	*0.002*
Albumin	1274	3,202±4,848	*0.002*
NGAL	1	9.9±5.4	*0.002*
Calbindin	2.84	21.8±13.0	*0.0039*
Clusterin	0.16	12.8±7.5	*0.0039*
Osteopontin	16	36.8±10.5	*0.002*

Supplementary Figure S1 | Not significative correlations of markers of acute kidney injury and eGFR at 1st, 3rd and 6th month after kidney transplantation.

Markers detected on perfusate do not correlate with moderate-term renal outcomes, in therms of renal function (eGFR).For each markers, patients of HOPE-K groups were divided based on median values.

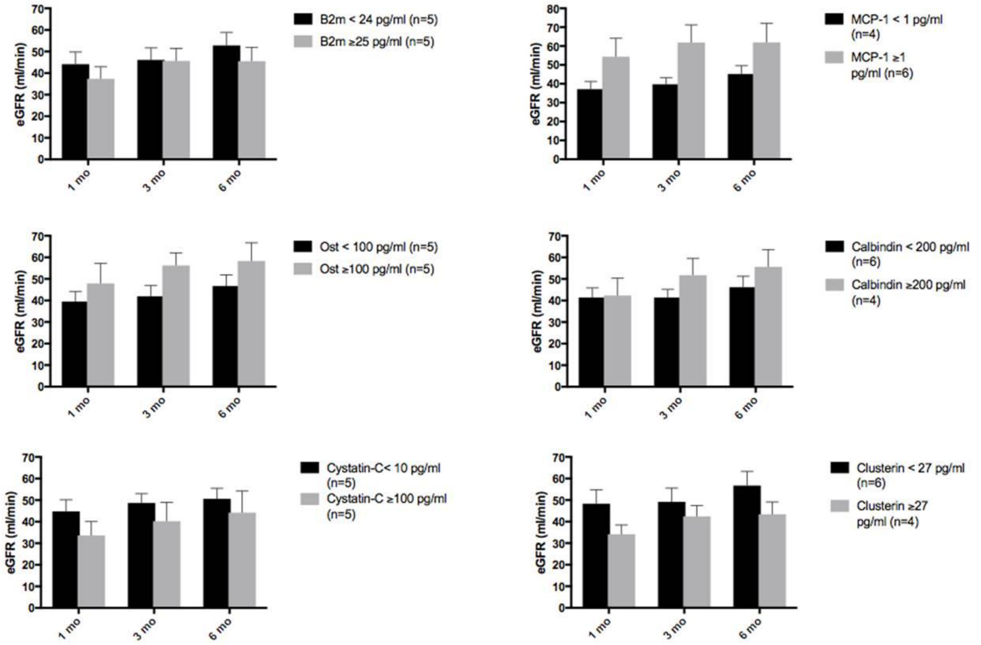

Supplement: Supplementary file 1 — Supplementary materials. [file 41598_2020_62979_MOESM1_ESM.pdf]
